# Hôtel Dieu

**Published:** 1832-12

**Authors:** 


### HOSPITAL REPORTS.

### HÔTEL DIEU.

*Clinical Lecture by* M. DUPUYTREN, *upon Cases of Removal of the Genital Organs.* (*Gaz. Médicale*, t. 3, No. 101.)

M. DUPUYTREN observed, that this kind of mutilation might depend upon various causes. Scarcely a year passed without patients being admitted who had lost their testicles by accidental violence. A case of this kind had lately been under the care of M. BRESCHET. The man had been deprived of one testicle in the following manner: he was unloading a waggon, and was accidentally caught by an iron hook, which lacerated the scrotum, and tore away one testicle. No hemorrhage ensued, and he was quickly well again. M. D. had known the testicles torn away by the teeth during a violent quarrel; and the still more extraordinary fact had fallen under his observation, of the testicles being torn away by the hands of a furious adversary. More frequently this mutilation is inflicted by the individual himself, during the existence of fever with delirium; and M. Dupuytren had admitted patients into the Hôtel Dieu, who had, whilst delirious, lacerated the scrotum either with bad knives or their nails, and removed the testicles. Sometimes madmen believe their safety depends upon the loss of their genital organs, and at other times weak-minded persons inflict this injury upon themselves for the most absurd reasons. One man removed his testicles to correct his daughter, who was addicted to habits of debauchery; another determined to play his wife a trick, by cutting off his penis. This last patient was prevented from completing his purpose, and he had only divided the urethra and a part of the corpus cavernosum. He was taken into the Hôtel Dieu, and an attempt was made to reunite the divided parts, a catheter having first been passed into the urethra. Union of the parts did take place, and, while in a flaccid state, the penis served very well to carry off the urine; but, during erection, as the cicatrix in one of the cavernous bodies prevented it, only one side of the organ was distended, and thus a very "hideous deformity" was produced. M. Dupuytren stated that, about twenty years ago, many cases were admitted of men who had lost one or both testicles: they were the victims of a wretched gelder, who travelled over the country announcing a radical cure for hernia!

The above remarks arose in consequence of the occurrence of a still more atrocious fact than any above mentioned; a repetition, in fact, of the dreadful atrocity formerly inflicted upon Abelard. The following is the case.

*Removal of the Right Testicle; Cure. Violent Removal of the Left Testicle eight weeks afterwards; Hemorrhage arrested by a Veterinary Process.*

Constantine M., æt. twenty-four, a workman in a foundry at Villette, was admitted, October 14th, with bleeding from the scrotum. The left side of the scrotum was distended, and of a violet colour: it was about the size of two fists. It had been divided by a neatly-made longitudinal incision, between the edges of which a large clot of blood projected; and from this there was a free and constant dripping of blood. It was not a true coagulum, and attempts were made to remove it in vain: it was blood infiltrated into the cellular tissue, as in a sponge. The edges of the wound were separated, and the cellular tissue, which was infiltrated to the thickness of a finger, was seized by forceps, and cut off by scissors. Three small arteries were secured, and the hemorrhage ceased.

In making the necessary examination of the parts, it was discovered that there was no testicle on the left side. The three vessels that had been secured did not proceed from the spermatic cord. The extremity of the cord was found to be pressed between two small pieces of wood tied together at each end, and very neatly applied, exactly in the same way as gelders and veterinary surgeons have recourse to in castrating animals. These circumstances appeared very extraordinary, and the surgeon was still more surprised on detecting, on the right side of the scrotum, a well-formed cicatrix, and that here also the testicle was wanting!

The cause of this double mutilation was as yet a mystery. The patient gave three or four different accounts, but all of them were very absurd. He was pressed with repeated questions, and his neighbours were likewise interrogated, and the following is the information that was at length obtained. This young man had cohabited with a married woman: her husband surprised him, and removed his right testicle, by way of a little salutary correction; and hence the first cicatrix, which had not been formed more than six or eight weeks. The patient said that he had been seized by four men, and, five days after the receipt of the injury, he repaired to the Hospital St. Louis for assistance, stating that he had met with his misfortune by accident. The unfortunate fellow, however, was no sooner cured, than he again commenced his intrigue with the same female, and, after having passed the night with her, her husband suddenly entered with two men: they tied his hands behind his back, and secured his legs by tying them to a bed-post. One of the wretches placed his knee on his chest, and his hand on his mouth, while the others executed their horrible purpose. The patient was too weak to offer any effectual resistance. The incision was very neatly made, and neither too large nor too small, and the man said it had been made with a small knife. The operator had been on his guard against hemorrhage from the spermatic cord; but his science went no further, for it appeared he had never thought of the arteries which alone furnished the hemorrhage. The bleeding from the cord was prevented by a neatly made and well-applied instrument, well known to veterinary surgeons; and M. Dupuytren believed, from the skill of the operator in one part of the operation, and from his ignorance in another, that he was a gelder by profession, or some person acquainted with the veterinary art.

The patient went on well, and the hemorrhage did not return; his generative powers were, of course, destroyed. Particular cautions were given that he should not be agitated by questions concerning his misfortune, and every means were suggested that were likely to dispel his depressed state of mind. He has since stated that the husband of his chere amie began the second operation, but that it was finished by some other person. He did not know the person who performed the first operation.

By a singular coincidence, another not less remarkable case is at present in the St. Marthe's ward of the Hôtel Dieu. The subject of it is a man aged forty-five, with black beard and moustaches. He entered with fever, but was sent by his physician to the surgeons. The scrotum, the testicles, and penis had been entirely cut away. A median and deep cicatrix gave to the small remains of the skin of the scrotum an appearance very much resembling the female labiæ. Instead of the penis, there was a small cicatrix, in the middle of which was a reddish-looking nipple, projecting about a line, and pierced by a hole about the size of a pin's head: this was the urethra. No account could be obtained from the patient as to the manner in which he had been thus mutilated. He first said he had been kicked by a cow; but he afterwards stated that, having lost, on the 5th of August, 1830, a sum of two thousand francs, by the fraud of a creditor, he had determined to destroy himself. He remembered that a physician, whom he named, had committed suicide by cutting off his penis and testes, and he followed the example. In this case there had been considerable hemorrhage, which had, however, yielded to the internal and external use of cold water. The patient afterwards treated and cured himself.

M. Dupuytren made an important remark in reference to the above case. When hemorrhage thus proceeds through extravasated blood, there are no other means of arresting it than by laying bare the vessels, and tying them; and the vessels can only be exposed by cutting through the infiltrated cellular tissue.

---

### *Clinical Lecture by* M. Dupuytren, *on Retraction of the Fingers.* (*Ibid.*)

M. Dupuytren, several months ago, directed the attention of his pupils to the subject of permanent flexion of the fingers, and the different causes of the deformity. Writers, in general, have only treated of that species of flexion of the fingers which is occasioned by hardened cicatrices from burns on the palm of the hand. M. Boyer, in his work on Surgery, briefly refers to the subject, and attributes the retraction of the fingers to a *crispatura tendinum*. The real causes and proper treatment of such cases were unknown, until M. Dupuytren discovered that the presumed contraction of the tendons was nothing more than contraction of the palmar aponeurosis, the division of which would immediately remove the deformity. At this time there was every reason to believe that this affection of the aponeurosis was accidental; the causes of it appeared evident, inasmuch as it existed usually in persons who were obliged to make frequent and violent efforts with the palm of the hand. Additional facts, however, have shewn that this contraction of the aponeurosis may be not only congenital, but also hereditary. In July, M. Dupuytren was consulted in the case of a child, six years of age, who had a permanent retraction of the little and ring fingers. There were no traces of cicatrix or burn; but, when the fingers were forcibly bent backwards, the extended aponeurosis raised the skin like a tight cord, and left no doubt as to the diagnosis. In this instance the deformity was congenital; the grandmother of the child had also been born with the same deformity. But, independent of retraction of the aponeurosis, and cicatrices upon the palm of the hand, other causes may also give rise to rigid contraction of the fingers, and different modes of treatment may be required. M. Dupuytren, for example, enumerates white swelling upon the joints of the fingers, and their consequent anchylosis; deformity of the articular surfaces from the effect of certain trades; the existence of serous cysts in or near the articulations; wounds of the flexor tendors; and paralysis of the extensor muscles. A case recently occurred at the Hotel Dieu from a different cause. The following is a brief account of it.

*Retraction of the Middle Finger, in consequence of Adhesions of the Extensor Tendon. Division of the Tendon; Cure.*

Virginie Lecord, three years old, had, at the age of a year, several small abscesses upon the lower third of the back part of the forearm. The two cicatrices that remained appeared too large for simple boils; but whatever might have been the nature of the abscesses, there was a cicatrix on the lower part of the forearm, outside the extensor tendon of the fingers, and a second inside the tendon, about half an inch lower down. The tendon formed a projection under the skin like a hard and rigid cord; the hand was bent backwards so as to form an obtuse angle with the back part of the forearm. The fingers were habitually in a flexed position; and it was only for a short time that the child had gained the power of voluntarily extending them. The state of the middle finger was different from that of the others; the first phalanx of it being bent much more backwards than the others; whilst the two other phalanges were rigidly flexed. In producing extension by force, the third phalanx was extended upon the second, but the latter remained obstinately bent upon the first.

M. Dupuytren regarded this as a very remarkable fact: the cause of the flexion existed neither near the joints nor the palm of the hand, but upon the back part of the forearm. There was no difficulty in explaining this apparent anomaly. In consequence of inflammation of the neighbouring parts, there were morbid adhesions and retraction of the extensor tendon, which satisfactorily explains the immoderate extension of the hand and first phalanx. But this forcible extension, this bending of the hand backwards, produced an unusual elongation of the flexor tendons and muscles, which were constantly endeavouring to regain their natural length; hence the habitual flexion of the fingers; but the extension being more powerfully exerted upon the middle finger, and pulling backwards the first phalanx, the action of the flexors, which could only act upon the two other phalanges, bent them inwards with still greater force. Every one may ascertain the validity of this explanation upon his own person. When all the muscles are in a state of repose, the fingers are more or less flexed, and they cannot be extended without bringing the flexor muscles into a greater or less degree of tension. If extension is forcibly produced, the tension of the flexors is augmented, and there is a degree of extension at which the hand cannot be forced further backwards upon the wrist, without flexion of the phalanges occurring in spite of any voluntary efforts to prevent it.

In the above case, the treatment, said M. Dupuytren, was obvious. It was necessary to divide the bridles which held the extensor tendon, and prevented it from yielding to the power of the flexor muscles, and perhaps also to divide the tendon itself. This division was made in the simplest manner; the tendon was exposed and cut across, and a part of the bridle, which was formed of a fibrous elastic tissue, was removed. The hand was immediately restored to a state of moderate extension; the middle finger was bent back, and the parts were maintained in position by an appropriate apparatus.

In fifteen days the little patient was discharged from the hospital entirely cured of the deformity. M. Dupuytren suggested that other causes would be discovered of this affection. It is very possible that the flexor muscles might be attacked with spasm, analogous to that which sometimes affects the sterno-mastoidean, and which necessarily draws down the head, and forcibly raises one shoulder. Some authors have pointed out a flexion of the finger as a symptom of luxation of the wrist, but Mr. Dupuytren doubts the existence of this injury.

